# Opening a can of worms: Archived canned fish fillets reveal 40 years of change in parasite burden for four Alaskan salmon species

**DOI:** 10.1002/ece3.11043

**Published:** 2024-04-04

**Authors:** Natalie Mastick, Rachel Welicky, Aspen Katla, Bruce Odegaard, Virginia Ng, Chelsea L. Wood

**Affiliations:** ^1^ School of Aquatic and Fishery Sciences University of Washington Seattle Washington USA; ^2^ Yale Peabody Museum Yale University New Haven Connecticut USA; ^3^ Department of Arts and Sciences Neumann University Aston Pennsylvania USA; ^4^ Unit for Environmental Sciences and Management North–West University Potchefstroom South Africa; ^5^ Seafood Products Association Seattle Washington USA

**Keywords:** Alaska, anisakids, canned fish, historical ecology, parasites, salmon

## Abstract

How has parasitism changed for Alaskan salmon over the past several decades? Parasitological assessments of salmon are inconsistent across time, and though parasite data are sometimes noted when processing fillets for the market, those data are not retained for more than a few years. The landscape of parasite risk is changing for salmon, and long‐term data are needed to quantify this change. Parasitic nematodes of the family Anisakidae (anisakids) use salmonid fishes as intermediate or paratenic hosts in life cycles that terminate in marine mammal definitive hosts. Alaskan marine mammals have been protected since the 1970s, and as populations recover, the density of definitive hosts in this region has increased. To assess whether the anisakid burden has changed in salmonids over time, we used a novel data source: salmon that were caught, canned, and thermally processed for human consumption in Alaska, USA. We examined canned fillets of chum (*Oncorhynchus keta*, *n* = 42), coho (*Oncorhynchus kisutch*, *n* = 22), pink (*Oncorhynchus gorbuscha*, *n* = 62), and sockeye salmon (*Oncorhynchus nerka*, *n* = 52) processed between 1979 and 2019. We dissected each fillet and quantified the number of worms per gram of salmon tissue. Anisakid burden increased over time in chum and pink salmon, but there was no change in sockeye or coho salmon. This difference may be due to differences in the prey preferences of each species, or to differences in the parasite species detected across hosts. Canned fish serve as a window into the past, providing information that would otherwise be lost, including information on changes over time in the parasite burden of commercially, culturally, and ecologically important fish species.

## Graphical abstract

Parasitic nematodes of the family Anisakidae (anisakids) can use salmon fishes as intermediate hosts in life cycles that terminate in marine mammal definitive hosts. To assess whether the anisakid burden has changed in salmon over time, we dissected a novel data source: chum, coho, pink, and sockeye salmon that were caught, canned, and thermally processed for human consumption in Alaska, USA between 1979 and 2019. Anisakid burden increased over time in chum and pink salmon, but there was no change in sockeye or coho salmon, possibly due to differences in the prey preferences of each species, or time spent in marine systems.

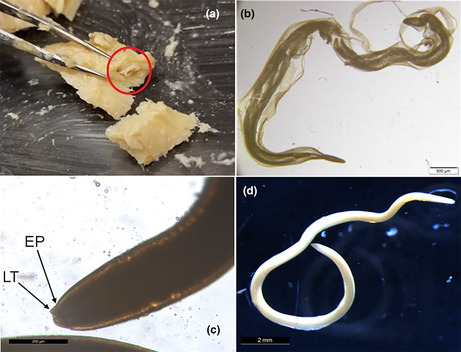

## 1 INTRODUCTION

Species that are difficult to study, including small and inconspicuous species like parasites, can experience changes in abundance that go entirely unnoticed. For example, between 1930 and 2016 a highly visible parasite of English sole (*Parophrys vetulus*) in Puget Sound, Washington, USA, *Clavinema mariae*, experienced an 8‐fold increase that went undetected for almost a century, even though sole were the subject of both a major recreational fishing industry and government research (Howard et al., 2019). For marine parasites, there are few data sources that can be used to reconstruct information about past abundances (Harmon et al., 2019; Wood & Vanhove, 2023). Comparing contemporary ecosystems to well‐studied ecosystems of the past by sampling the same sites with the same methodology has been effective (e.g., Quinn et al., 2021), but is constrained by the available data, which is contingent upon the interests of past researchers, sufficient documentation, and data retention. Local and Indigenous ecological knowledge can extend historical baselines farther back in time (e.g., Jessen et al., 2022), and in some cases can provide insights into changes in parasite abundance (Tomaselli et al., 2018), but to our knowledge such methodology has yet to be successfully applied to parasites in marine systems, and so associated limitations are not well‐understood. Meta‐analyses of existing scientific literature can be a useful tool to detect trends in parasite abundance (e.g., Fiorenza, Wendt, et al., 2020), but are limited by the temporal constraints of online literature repositories (Wood & Vanhove, 2023). Natural history collections are a promising source for reconstructing parasite population data (Fiorenza, Leslie, et al., 2020; Harmon et al., 2019; Howard et al., 2019; Welicky et al., 2021; Wood, Leslie, et al., 2023; Wood, Welicky, et al., 2023), but their use is limited to what host species are available in collections.

Limitations on the techniques, datasets, and resources available to reconstruct long‐term trajectories of parasite abundance are a cause for concern because parasites are influential in ecosystems. Parasites can affect host populations and the communities and ecosystems in which they are embedded (Hudson et al., 2006; Lafferty, 2008; Wood & Johnson, 2015) and as a result, they can pose a threat to fisheries (Lafferty et al., 2015). It is particularly important to understand how the burden of parasitism is changing for economically and commercially important hosts, given that parasites can influence these populations (Lafferty et al., 2015; Shinn et al., 2015; Timi & Poulin, 2020). Salmon (*Onchorhynchus* spp.) are culturally, economically, and ecologically important in Alaska (Alaska Department of Fish and Game, 2023; Carothers et al., 2021), with an ex‐vessel commercial value of $786 million in 2021 (Alaska Department of Fish and Game, 2023). Salmon also make up a large part of the diet of many marine and terrestrial predators (Helfield & Naiman, 2006; Morton, 1990; Quakenbush et al., 2015; Sigler et al., 2009; Stanek et al., 2017). An increase in parasites in salmon could impact Alaskan residents and wildlife, as well as the state's economy, but without historical data on the parasite burden in salmon, change is not quantifiable.

Unfortunately, there are few pathways for quantifying change in the parasite burden of salmon. Salmon are common intermediate hosts to parasitic nematodes of the family Anisakidae, or anisakids (Deardorff & Kent, 1989). These parasites have complex life cycles: after hatching in the ocean, anisakids have a brief free‐living stage, which is consumed by a zooplankton intermediate host, which is consumed by a fish (including salmon) or cephalopod intermediate host before reaching their definitive host, a marine mammal (e.g., Klimpel & Palm, 2011). Infections in the musculature of fish can inhibit swimming ability and increase susceptibility to predation, as well as cause other pathogenic effects (Buchmann & Mehrdana, 2016). Should anisakid infections be increasing, this could add to the list of stressors that salmon face, including habitat loss and degradation (Schoen et al., 2017), climate change (Bryant, 2009), and increasing predation from recovering marine mammal populations (Chasco et al., 2017). However, we can identify no long‐term datasets of parasite abundance for any adult salmon species from anywhere in the world (Fiorenza, Wendt, et al., 2020); the longest‐term datasets on parasites in salmon that exist include parasites in sockeye salmon smolts (41 years; Bennett et al., 1998), parasites in juvenile sockeye salmon (12 years; Bentley & Burgner, 2011), and sea‐louse infestations on juvenile pink and chum salmon (22 years; Peacock et al., 2016), but these datasets do not reflect the burden of endoparasites in adult salmon from marine systems. Museum specimens of adult salmon are rare, as they would occupy a substantial amount of space in space‐limited collections (Wood, Leslie, et al., 2023). Processors sometimes record parasite presence, especially nematodes in the musculature, but these data are usually not stored for more than a few years (Joe Logan, Alaska Seafood Industry Professional, personal communication). Meta‐analysis would be an option, but there are few papers on salmon parasites in the Northeast Pacific, where many native salmon runs exist (Fiorenza, Wendt, et al., 2020). For salmon, because many of these methods of historical parasite reconstruction are unavailable for the reasons mentioned above, changing parasite burden cannot be quantitatively assessed.

Propitiously, we came across a data source maintained by an industry organization that could be used to detect a change in parasite burden for salmon in the Northeast Pacific: over four decades of canned Alaskan salmon, preserved to determine how can integrity was maintained over time. Four species of salmon were canned consistently across the Gulf of Alaska and into Bristol Bay from 1979 to 2021: chum (*Oncorhynchus keta*), coho (*Oncorhynchus kisutch*), pink (*Oncorhynchus gorbuscha*), and sockeye (*Oncorhynchus nerka*). We were able to find anisakid worms in this material, providing a novel data source to test whether there has been a change in anisakid burden in salmon over the past 42 years.

Many factors could affect anisakid abundance over time. Climate change could impact complex life cycle parasites negatively (Carlson et al., 2017; Wood, Welicky, et al., 2023), or positively, as many anisakids are resilient to warming temperatures (Measures, 1996). Increasing abundances of some marine mammal definitive hosts (Ford et al., 2009; Lowry et al., 2008; Matkin et al., 2014; Muto, 2021; Towers et al., 2015; Towers et al., 2019) could increase anisakid prevalence in salmon (Buchmann & Kania, 2012; Haarder et al., 2014; Zuo et al., 2017). We expected the magnitude of the change might differ by salmon species, as salmon species have distinct life histories and habitats that could result in differing levels of exposure (Brodeur et al., 2007; Johnson & Schindler, 2009; Kaeriyama et al., 2004).

We dissected canned salmon samples and extracted, counted, and morphologically identified any nematodes present, acquiring a measure of anisakid abundance per gram of fillet. We then ran generalized linear mixed‐effect models to determine if anisakid abundance had changed over time for each salmon species. Our study is the first to use archival canned salmon to detect and quantify changes in parasite abundance over time.

## 2 METHODS

There are several constraints to using historical data, including biases and inconsistencies in reporting, as well as temporal and spatial gaps or patchiness (McClenachan et al., 2012, 2015; Swetnam et al., 1999). However, these shortcomings do not invalidate the data (McClenachan et al., 2015; Swetnam et al., 1999), provided that any biases are recognized and addressed (McClenachan et al., 2015; Swetnam et al., 1999). To our knowledge, this paper is the first to use canned salmon for historical reconstruction of past parasite abundance. To address the biases related to this dataset, we needed to account for potentially confounding variables, including the location of the cannery, chilling practices used on board vessels, and fishing region.

### 2.1 Selection and dissection of archived specimens

**FIGURE 1:**
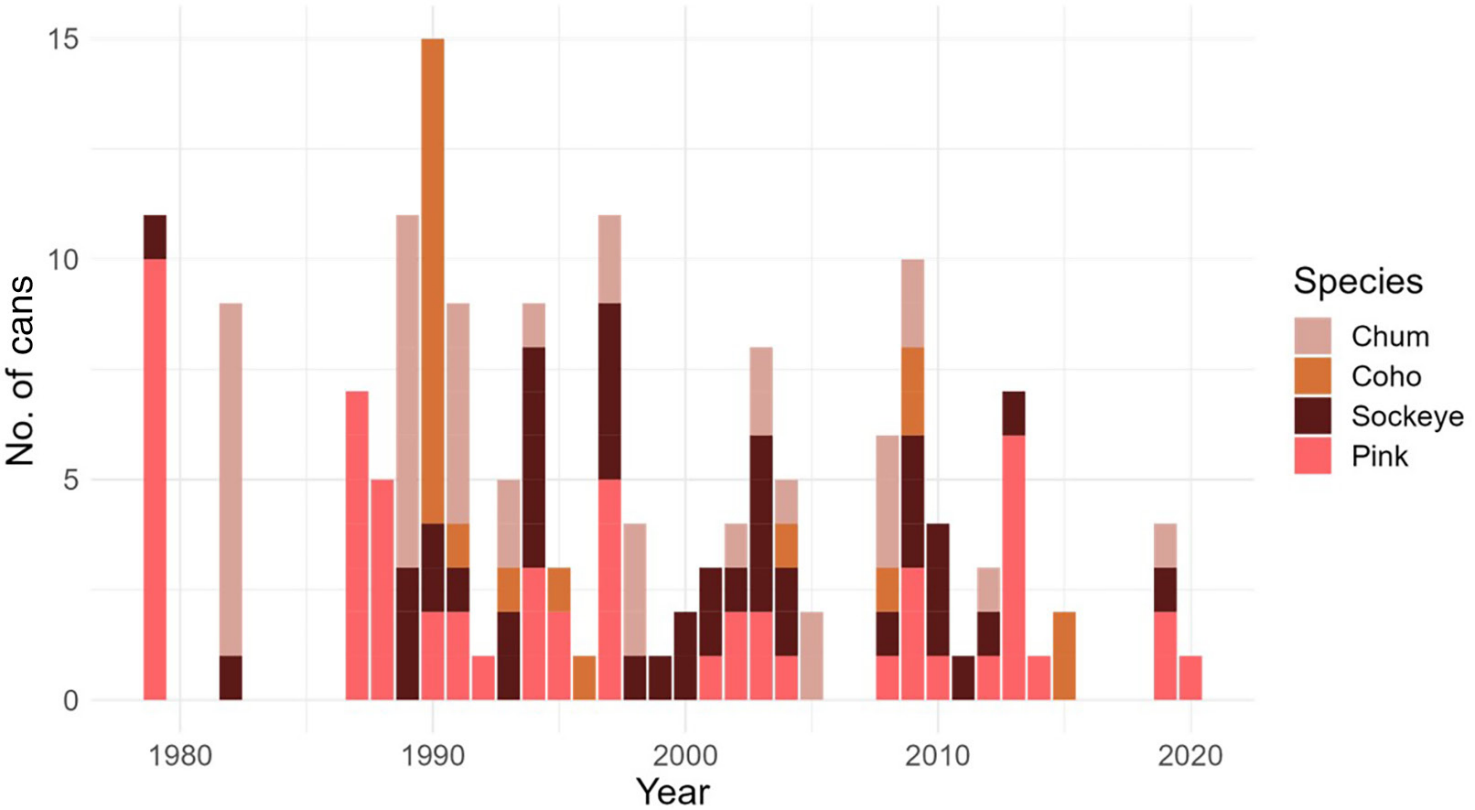
The distribution of canned salmon samples available for each salmon species in each decade.

In 2020, BO and VN approached CLW, NM, and RLW to ask whether the University of Washington research team might be able to detect parasites in an extensive collection of cans amassed by the Seafood Products Association, an industry association which BO and VN were leading. The Seafood Products Association had retained samples of products from the 1970s onward for the purpose of assessing the degradation of canned products, but in 2020 they learned about the historical parasite ecology studies our lab was conducting and offered cans to us. From this collection, BO identified 502 cans of salmon, including fillets from chum, coho, pink, and sockeye salmon originating from Alaska and Washington, produced by various companies and canneries, and canned between 1979 and 2021. There were very few cans from Washington, so we decided to focus exclusively on Alaska. Our goal was to analyze 15 cans per species for each decade. We selected cans from well‐represented cannery locations when available (i.e., more than 1 can available from that location). For coho, there were few can samples available, so all cans available were used for analysis. We did not include cans that had unknown dates of packaging, or unknown species contents. Some cans did not include a specific year, and were only classified as “pre‐1982”, and these were excluded from analysis. After these exclusions, our dataset contained pink (*n* = 62), sockeye (*n* = 52), chum (*n* = 42), and coho (*n* = 22) cans spread as evenly as possible across decades (Figure 1). Can sizes varied, including cans that were 3.75, 7.5, and 14.75 oz. Due to the degradation of some of the fillets into the liquid in the cans, it was difficult to determine the mass of the fillets. Therefore, we did not weigh the salmon fillet; instead, we accounted for fillet size with the can size converted to grams. Additionally, it is possible that multiple fish could be included in a single can, and a single fish could have been used to fill multiple cans (Bruce Odegaard, Seafood Products Association, pers. comm.).

**FIGURE 2:**
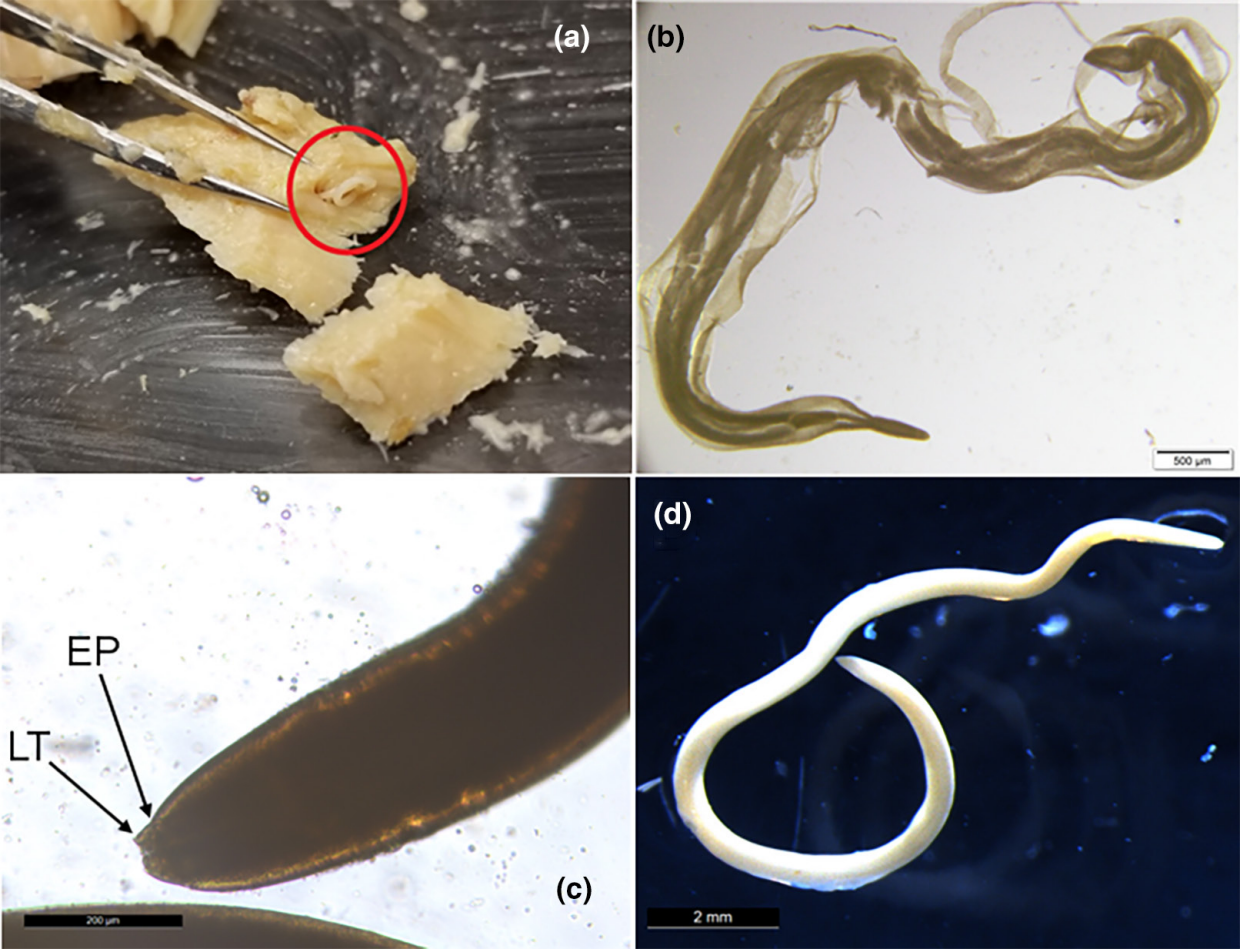
(a) A photo of a nematode (red circle) in a canned salmon fillet. The nematodes coil within the muscle and form pockets that are easily detected when dissecting with forceps. (b) A nematode recovered from canned salmon, cleared with lactophenol solution. Nematodes were highly degraded during the canning process, to the extent that even clearing the recovered specimen could not give us sufficient information to form an accurate genus‐level identification. (c) An ethanol‐preserved, uncleared anisakid nematode, was identified at the family level by the presence of a larval tooth (LT) and an excretory pore (EP) ventral to it, as described by Hurst (1984). (d) A preserved, uncleared anisakid. The cuticle is very cloudy, and internal organs are not visible.

After method testing (Appendix S1), we found that dissecting the fish into small pieces with two, 4.25‐inch fine‐point forceps was the most efficient way of detecting and extracting nematodes. Nematodes formed pockets in the musculature of the fillet, which were easily detectable as we dissected (Figure 2a). When we found a nematode, we carefully extracted the worm and preserved it in 70% ethanol. We validated our detection method by having a second observer check the work of the dissector until we achieved 100% agreement, after which we chose 6% of cans at random to check to ensure consistent agreement. Fillets in older cans were more fragile, as degradation of the fillet had occurred over time, but nematodes were still easily detectable.

We expected that all nematodes detected in the fillets would belong to the family Anisakidae because anisakids are the only parasite family known from the muscle of Alaskan salmon (Moles, 2007). In past studies in the Puget Sound, salmon hosts have had a high prevalence of *Anisakis simplex* – reaching 100% prevalence in sockeye (Deardorff & Kent, 1989) and chum (Myers, 1979). Similarly, in Bristol Bay and Prince William Sound, Alaska, all chum, pink, and sockeye salmon sampled were found to have *Anisakis simplex* infections (Karl et al., 2011). We attempted to confirm the identity of the nematodes to the genus level in two ways: (1) by clearing the vouchered nematodes with lactophenol solution according to standard nematode identification procedure (Cable, 1963) and identifying based on internal anatomy, and (2) by morphologically identifying the vouchered nematodes under a stereomicroscope using external anatomy only. Often, nematodes were broken or too tightly coiled to identify. The nematodes did not clear entirely (Figure 2b) and the cuticle remained cloudy, therefore the internal anatomy was difficult to identify. Therefore, we identified nematodes based on external anatomy where we were able. When the nematodes were not broken or too tightly coiled, we identified the worms as anisakids by the presence of a larval tooth and ventral excretory pore, which appeared as a dimple on the anterior end (Figure 2c), indicative of a nematode in the family Anisakidae (Hurst, 1984). We were able to definitively identify 34% (127/372) of the nematodes we found; of these, 100% were anisakids. Therefore, we assumed that all detected nematodes were anisakids, including those that could not be definitively identified.

### 2.2 Geographical predictors

**FIGURE 3:**
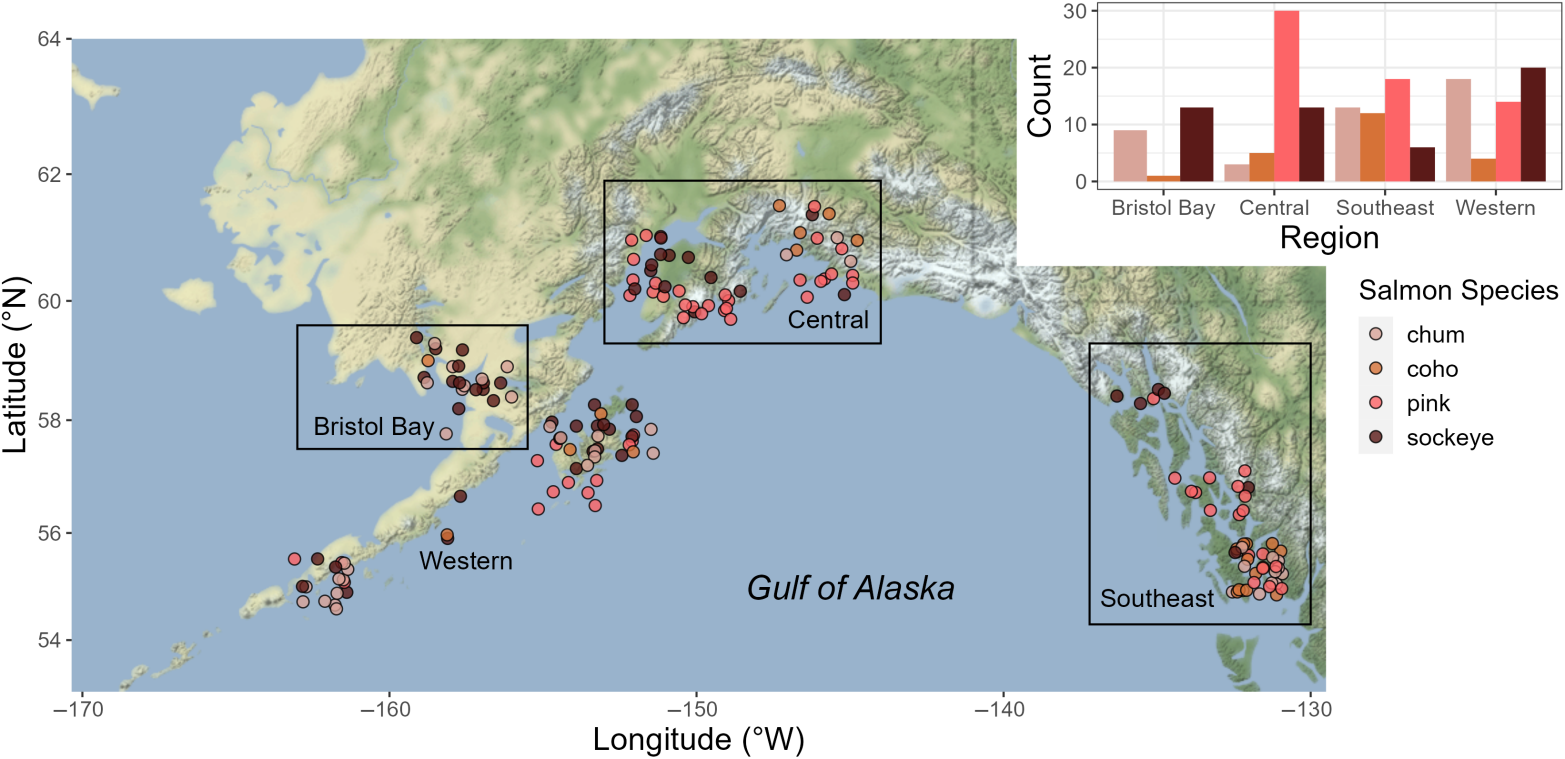
A map of the canning locations for each canned salmon sample, where each dot is a can that we dissected, color reflects the species of salmon canned (chum in light pink, coho in orange, pink in hot pink, and sockeye in burgundy), and the points are jittered to avoid overlap. The inset figure shows the number of cans of each species collected in each region. Most cans came from the Gulf of Alaska (Western, Central, and Southeast regions), though some chum, sockeye, and coho were collected from the Bristol Bay region.

To quantify differences in anisakid burden among geographical regions, we classified the region where each can be sourced, and therefore, where the fish was caught. We used four regions, adhering to the fishing regions commonly described by seafood companies in this area: Bristol Bay, which included all canneries north of the Aleutians; Western, which included canneries on the Aleutians over to Kodiak, AK; Central, which included canneries between Homer and Cordova, AK; and Southeast, which included canneries from Excursion Inlet south to Ketchikan, AK. To incorporate the cannery location as a proxy for the area of capture, we collected the latitude and longitude of the town where each cannery was located using Google Maps (Figure 3).

Transport and processing of salmon differs among species and over time and made it necessary for us to split our analysis into two statistical models: one for pink, chum, and coho salmon, and another for sockeye salmon. Pink, chum, and coho salmon decay quickly in transport, and are therefore almost always processed in the region where they are caught, typically within a 50‐mile radius of the cannery (John Daly, industry expert, pers. comm.). To account for this, we included in our statistical model for parasite burden in pink, chum, and coho salmon a random effect of region (see Section 2.4: Statistical analysis, below).

Conversely, sockeye is more robust to decay and therefore more transportable. Sometimes sockeye were canned in regions adjacent to where they were caught, though this varied by year of collection (John Daly, industry expert, pers. comm.). Sockeye in the more southern areas of their range faced declines from the 1990s to mid‐2000s (Rand et al., 2012), while Bristol Bay populations increased (Hilborn, 2006; Rand et al., 2012). This led to sockeye being transported outside of Bristol Bay to be canned in other regions, starting roughly in the 2000s (John Daly, industry expert, pers. comm.). In 2018, two major canneries closed in Bristol Bay, so fish caught in this region were more commonly shipped outside of Bristol Bay for canning (John Daly, industry expert, pers. comm.). Because of these differences in catch region, we created a time period variable representing periods in which transport patterns were consistent: pre‐2000s (local canning), 2000–2018 (some non‐local canning), and 2018–2021 (common non‐local canning). We included this variable in our statistical model for parasite burden in sockeye salmon, where the model calculated an intercept for the interaction between time period and region as a random effect (1 | region*time period).

### 2.3 Onboard chilling practices

By the mid‐1990s, all fishing vessels were strongly encouraged to have chilling capability onboard, usually ice or a tank of seawater cooled to 0–2.2°C degrees for vessels fishing near the cannery, and − 2.2°C for vessels transporting the catch a greater distance (John Daly, industry expert, pers. comm.). Not only does this keep the fish fresh longer, but it also may slow the migration of anisakids into the muscles of the fish (Cipriani et al., 2016). Anisakids can inhabit the viscera of intermediate hosts and often will attempt to escape the host when the host dies by migrating to the muscle (Smith & Wootten, 1975). The migration of anisakids from the viscera to the fillet may be tied to an increase in temperature, so reducing the temperature at which the fish is stored results in fewer nematodes in the fillet (Cipriani et al., 2016). To account for this change in the preservation method, we incorporated an additional fixed effect of the chilling method in our models (see Section 2.4: Statistical analysis, below). We did not know the exact date that chilling practices were adopted for each vessel but assumed that cans dated before 1995 did not have on‐board chilling, while cans during and after 1995 did, based on expert opinion (John Daly, industry expert, pers. comm.).

### 2.4 Statistical analysis

We tested whether nematode counts per can changed over time with generalized linear mixed effect models (GLMMs). First, we wanted to ensure that our statistical outputs would not be biased by spatial or temporal autocorrelation. We used a simplified model (anisakid count ~ year) and tested for spatial autocorrelation with the testSpatialAutocorrelation() function in the DHARMa package (Hartig, 2022) in R (R Core Team, 2021). We tested for temporal autocorrelation with a Durban‐Watson test using the dwtest() function in the lmtest package (Zeileis & Hothorn, 2002) in R. We used the glmmTMB() function in the package of the same name (Brooks et al., 2017) in R to run both GLMMs. We detected that the data from sockeye salmon were spatially autocorrelated, so we included regions in the corresponding model to account for spatial differences.

We fit a global GLMM and then compared it to several candidate models that tested alternative hypotheses (Tables S1 and S2). We determined which model was best supported by the data using AIC values. After model selection, we ran two GLMMs to determine how the number of anisakids changed over time. We ran two models to account for differences between the salmon species; since sockeye salmon were transported differently than chum, coho, and pink salmon (see above), we ran a separate model for sockeye salmon. We could not determine how many individual fish were in each can, so we quantified the number of anisakids per can as the response variable. We tested for the effect of can size because cans with a greater mass of salmon fillet should yield a higher anisakid count, on average.

Model 1 – tests whether anisakid abundance, the response variable, changed in coho, chum, and pink salmon, accounting for geographical, cannery, and management company differences, and incorporating can size and chilling practices. We used a negative binomial distribution to account for zero inflation in parasite counts that is commonly observed (Shaw et al., 1998). Can size and year were scaled using the scale() function in R. Scaled can size, chilling practice, and the interaction between scaled year and salmon species were included as fixed effects. Can size and year were continuous variables while chilling practice and salmon species were categorical. Factory and region nested within the company were included as random effects to account for variability due to geographical or commercial differences.

Model 1:

$$N{\text{Anisakids}}_{\text{ijkl}}\sim \text{Negative binomial}\;\left(\mu_{\text{ijkl}}\right)$$

$$E\left(N{\text{Anisakids}}_{\text{ijkl}}\right)=\mu_{\text{ijkl}}$$

$$\begin{array}{c} \text{the}\;\log\left(\mu_{\text{ijkl}}\right)={\text{ScaledCanSize}}_{\text{ijkl}}+{\text{ScaledYear}}_{\text{ijkl}}\times {\text{FishSpecies}}_{\text{ijkl}}\\ +{\text{ChillingPractice}}_{\text{ijkl}}+{\text{Factory}}_{j}/{\text{Region}}_{k}+{\text{Company}}_{l}/{\text{Region}}_{k} \end{array}$$

$${\text{Factory}}_{j}\sim N\left(0,\sigma^{2}\right)$$

$${\text{Region}}_{k}\sim N\left(0,\sigma^{2}\right)$$

$${\text{Company}}_{l}\sim N\left(0,\sigma^{2}\right)$$

where the response variable_ijkl_ represents a measurement of nematode abundance from the *i*th can from the *j*th factory where the sample was canned, in the *k*th region where the factory was located, by the *l*th company that the factory was run by.

Model 2 – the sockeye model, which is the same as Model 1 except that it incorporates the effect of region interacting with time period. Because sockeye were transported long distances for canning, and their factory location may not be representative of where they were caught, especially following the closure of two canneries in Bristol Bay, we needed to incorporate the time period (pre‐2000s, 2000–2018, and 2018–2021) into the model. Scaled can size, factory, company, and the interaction between region and time period (pre‐2000s, 2000–2018, and 2018–2021) were included as fixed effects.

Model 2:

$$N{\text{Anisakids}}_{\text{ijklm}}\sim \text{Negative binomial}\;\left(\mu_{\text{ijklm}}\right)$$

$$E\left(N{\text{Anisakids}}_{\text{ijklm}}\right)=\mu_{\text{ijklm}}$$

$$\begin{array}{c} \log\left(\mu_{\text{ijklm}}\right)={\text{ScaledCanSize}}_{\text{ijklm}}+S{\text{caledYear}}_{\text{ijklm}}+{\text{Region}}_{j}\\ \times {\text{TimePeriod}}_{k}+{\text{Factory}}_{l}+{\text{Company}}_{m} \end{array}$$

$${\text{Region}}_{j}\sim N\left(0,\sigma^{2}\right)$$

$${\text{TimePeriod}}_{k}\sim N\left(0,\sigma^{2}\right)$$

$${\text{Factory}}_{l}\sim N\left(0,\sigma^{2}\right)$$

$${\text{Company}}_{m}\sim N\left(0,\sigma^{2}\right)$$

where the response variable_ijklm_ represents a measurement of nematode abundance from the *i*th can from the *j*th region that the factory was located interacting with the *k*th time period of collection, from the *l*th factory, by the *m*th company.

## 3 RESULTS

We dissected 178 cans collected from Alaska from 1979–2021, including 42 cans of chum, 22 of coho, 62 of pink, and 52 of sockeye. Of these cans, 50.6% contained nematodes, including 57.1% of chum cans, 27.3% of coho, 46.8% of pink, and 59.6% of sockeye. A total of 372 nematodes were extracted from the cans. Among all nematodes detected, 37.7% (140/372) had a larval tooth consistent with the anatomy of nematodes of the family Anisakidae, and of those, 90.3% (127/140) had a visible dimple consistent with the location of the excretory pore on members of the family Anisakidae (Hurst, 1984). Of all nematodes extracted, 34% (127/372) were confirmed as belonging to the family Anisakidae by the identification of both a larval tooth and an excretory pore. The remaining nematodes were unidentifiable because they were broken (anterior end), the cuticle was too cloudy to identify morphological features, the nematode was too tightly coiled to identify the anterior end without damaging the specimen, or the vouchered specimen dried up.

### 3.1 Model 1

**FIGURE 4:**
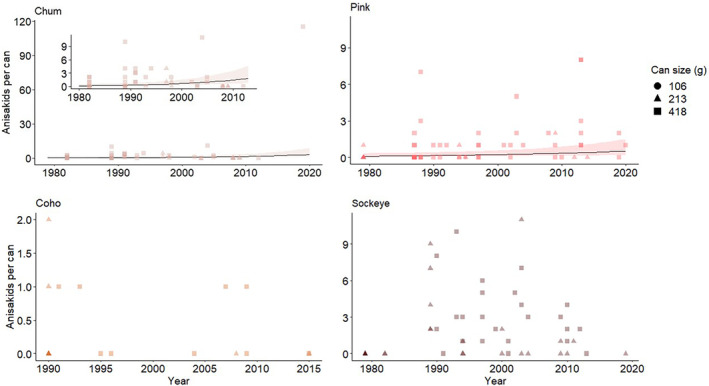
The predicted effect of time on anisakids for chum and pink, which experienced significant increases, in Model 1, and the raw data points for coho and sockeye, which did not experience a significant change over time. In chum and pink, predictions are based on the region with the most observations (Southeast), a random factory, the most sampled company, chilling practices in effect (i.e., post‐1995 group), and the median can size. However, the random effect of the region had minimal effect on the model predictions. Points represent raw count data jittered, which resulted in variability in the graphed points. The inset graph for chum is the same graph with axes truncated to exclude the high‐influence observation of 115 nematodes in 2019.

Three models fell within 2 ΔAIC of the top model. Our best‐fit model included scaled can size, and the interaction between salmon species and scaled year, excluding the effect of chilling practice (Table S1). The model with all of the factors above plus chilling practice as a fixed effect was the second best fit (ΔAIC = 0.3), and a candidate model testing the effect of year and salmon species as separate fixed effects was the third best fit (AIC = 1.9). Because we were interested in determining how anisakid counts changed over time for each salmon species, and we wanted to account for the potential effects of chilling practices on trends in parasite abundance, we ran the second‐best fit model. We found different trends in anisakid burden among host species (Table 1). Anisakids were estimated to be increasing in chum (estimate = 1.254, *p* = .004), and in pink salmon (estimate = 0.800, *p* = .015), but coho salmon did not display significant trends by year (Table 1, Figure 4). As we expected, there was an increase in the number of anisakids with increasing can size (estimate = 0.6112, *p* = .00357); the can size effect controlled for differences in the mass of fish tissue evaluated among cans. The chilling practice did not have a significant effect on the anisakid burden in the fillet.

**TABLE 1:** Results from Model 1, incorporating data from pink, coho, and chum, with significant effects are in bold.

Variable	Estimate	SE	*Z* value	*p* value
Can size (g)	0.611	0.210	2.914	**.004**
Chilling practice	0.789	0.593	1.330	.183
Year * Chum	1.258	0.306	4.115	**3.88e‐05**
Year * Coho	−0.191	0.752	−0.253	.800
Year * Pink	0.800	0.328	2.438	**.015**
**Random effects**	**Variance**	**SD**		
Factory	7.836e‐10	2.799e‐10		
Company/Region	8.067e‐10	2.84e‐05		

*Note*: We ran the analysis three times, changing the reference salmon species to obtain the estimate, standard error, *Z* value, and *p* value for each species. Random effect values were calculated with chum in the reference position.

While we detected an increase over time in chum, the trend was driven by a single count of 115 nematodes in a can from 2019. We were certain that this count was accurate, but it was also highly influential: removing this data point resulted in a non‐significant trend. We sought to understand the likelihood that this result arose due to random chance by running a permutation test on a simplified model of anisakid count ~ year for chum salmon data. For 5000 iterations, we randomly resampled the year without replacement for each data point, repeated the analysis, and recorded the estimate for the effect of the year. The resulting *p*‐value (i.e., the proportion of permutations with an estimate greater than the observed estimate) was 0.0136, indicating that it was statistically unlikely that the observed increase over time arose due to random chance.

### 3.2 Model 2

Following model selection, the best‐fitting model for sockeye did not include the year as a fixed effect (Table S2). This suggests that year is not an informative predictor for anisakid prevalence in sockeye salmon. The best fitting sockeye model included can size as a fixed effect and random effects of the interaction of region and time period, company, and factory ID. For the sockeye‐only model, there was a significant increase in the number of anisakids with increasing can size (estimate = 0.529, *p* = .0147; Table 2).

**TABLE 2:** Results for Model 2, with sockeye‐only data. Significant effects are in bold.

Variable	Estimate	SE	*Z* value	*p* value
Can size	0.529	0.217	2.439	**.015**
**Random effects**	**Variance**	**SD**		
Region*Time Period	0.335	0.579		
Factory	0.483	0.695		
Company	0.187	0.432		

## 4 DISCUSSION

We found a significant increase in anisakid burden in chum and pink salmon over the 42‐year study period. We found no significant change in the number of anisakids in coho and sockeye. The increase in anisakids we detected in chum and pink salmon in this study agrees with observations from other systems. Meta‐analysis of fish and cephalopod species has detected a global increase in *Anisakis* spp. in intermediate hosts since the 1960s (Fiorenza, Wendt, et al., 2020; Mastick, Fiorenza, et al., 2024). More locally, parasitological dissection of museum specimens revealed a recent increase in another anisakid, *Contracaecum* spp., in Puget Sound fish, following a precipitous decline from 1920–1995, probably due to fluctuations in harbor seal abundance (Mastick, Welicky, et al., n.d.). Though our study made use of a novel data source, the trend detected is consistent with previous studies from other regions (Fiorenza, Wendt, et al., 2020; Mastick, Fiorenza, et al., 2024), and reveals a previously undetected increase in anisakid nematodes in some species of Alaskan salmon over the past 40 years.

### 4.1 What could drive an increase in anisakids?

The increase we observed in parasite burden across time may reflect an increase in one or more definitive host species. Marine mammals are the definitive hosts of anisakids, and these species have had varying population trends throughout Alaska. While there have been regional declines in Cook Inlet beluga whales (*Delphinapterus leucas*) and the western stock of Steller sea lions (*Eumetopias jubatus*), most species have been increasing in Alaskan waters since the implementation of the Marine Mammal Protection Act in 1972, including: Northern fur seals (*Callorhinus ursinus*) on Bogoslof Island, harbor seal (*Phoca vitulina*) populations in Bristol Bay and Kodiak (Muto, 2021), humpback whales (*Megaptera novaeangliae*), Bristol Bay belugas, southern Alaska resident killer whales, Northern resident killer whales, and West Coast transient killer whales (*Orcinus orca*; Ford et al., 2007; Lowry et al., 2008; Matkin et al., 2014; Muto, 2021; Towers et al., 2015, 2019).

With increases in marine mammal hosts, there can be corresponding increases in the anisakids detected in intermediate fish hosts. Such trends have been observed in the Baltic Sea with gray seals and Baltic cod (Buchmann & Kania, 2012; Haarder et al., 2014; Mehrdana et al., 2014), and in Puget Sound with harbor seals (Mastick, Welicky, et al., n.d.). In the Baltic Sea, an increase in gray seals (*Halichoerus grypus*) resulted in an increase in the prevalence of Baltic cod (*Gadus morhua*) of *Contracaecum* spp. (prevalence = 22% in 1980s, 55.1% in 2012, 100% in 2014) and *Pseudoterranova* spp. (prevalence = 2% in 2011, >50% in large cod and 20% in small cod in 2014; Haarder et al., 2014; Mehrdana et al., 2014). In Puget Sound, WA, USA, there has been a recent increase in the number of *Contracaecum* spp. found in five fish species, possibly due to increasing harbor seal abundances in the area (Mastick, Welicky, et al., n.d.). As juveniles, pink and chum salmon spend several months living in nearshore marine and estuarine regions before moving offshore (Howard et al., 2017; Levings, 2016). Coastal regions and estuaries in Alaska are frequently used by marine mammals, including pinnipeds, belugas, and humpback whales (Muto, 2021), which could increase the prevalence of anisakids in the environment. This could result in an increasing risk to salmon of exposure to infected prey if salmon feed more prevalently in coastal areas nearby seal haulouts or areas frequented by marine mammals.

The change over time we observed could also be attributable to increases in sea surface temperatures (SSTs). Though climate change could have negative effects on complex‐life cycle parasite transmission (Carlson et al., 2017; Marcogliese, 2008; Wood, Welicky, et al., 2023), there are some scenarios in which anisakid prevalence could increase with increasing temperatures. Warming temperatures could increase parasite abundance by increasing parasite vital rates or decreasing host immunity (Claar & Wood, 2020; Macnab & Barber, 2012). Increased temperatures have been found to speed up the development of anisakid eggs (Measures, 1996). Anisakids are generally protected from changing environmental conditions by their hosts, but they have a brief, free‐living stage in the marine environment (Klimpel & Palm, 2011). During this phase, their protective cuticles allow them to survive in a variety of environmental conditions (Page, 2013). For one species, *Pseudoterranova decipiens*, though the optimal temperature for larval survival is 5°C, they can survive up to 45 days in 15°C temperatures (Measures, 1996), which is slightly greater than the average annual SST highs in Alaska (National Centers for Environmental Information (NCEI), 2023). However, this narrower window of survival could result in reduced temporal overlap between anisakid larvae and their requisite hosts (Paull & Johnson, 2014). Additionally, climate change will impact the distribution of anisakid hosts (Marcogliese, 2008). Even if climate change negatively impacts some intermediate host species in Alaska, as ocean temperatures in northern latitudes increase, warm water hosts may move northward (Marcogliese, 2008). This range expansion is expected to increase the prevalence of anisakids in northern regions (Klimpel & Palm, 2011). An increase in growth rates and an expanded geographic range of anisakid hosts with increasing temperatures could have contributed to the increase we observed in anisakid infections over our study period, although it does not explain why we observed increases in some salmon species but not others.

The salmon species we assessed are opportunistic foragers but have different diets (Brodeur et al., 2007; Johnson & Schindler, 2009; Kaeriyama et al., 2004). Coho is piscivorous, while chum, pink, and sockeye feed on lower trophic level prey, consisting mostly of zooplankton (Brodeur et al., 2007; Johnson & Schindler, 2009; Kaeriyama et al., 2004). Salmon diet shifts annually with variability in environmental conditions (Kaeriyama et al., 2004), and may change with increasing temperatures (Beamish et al., 2004; Coyle et al., 2011). In the Bering Sea, pink, chum, and sockeye have shifted their diets in periods of warming temperatures, consuming more fish in warm years and more euphausiids in cooler years (Coyle et al., 2011). If the fish preyed upon are at a trophic level higher than euphausiids, they may be more likely to have accumulated anisakid parasites (Lester & McVinish, 2016). In the Strait of Georgia, rather than a diet shift, warmer periods resulted in increased feeding intensity and frequency, as well as increased size and survival (Beamish et al., 2004). Larger fish in general have more parasites (Poulin, 2000), and parasite biomass increases linearly with host mass (Poulin & George‐Nascimento, 2007). In addition, fish in better condition, quantified by a combination of mass and length, have been shown to have higher parasite loads (Lagrue & Poulin, 2015). An increase in feeding intensity could increase the likelihood of consuming infected prey, which would increase anisakid prevalence in salmon feeding in marine systems.

An increase in feeding intensity alone does not explain the different trends we detected among host species, which could arise if there are multiple species of anisakid with different host affinities (e.g., Klimpel & Palm, 2011) and different abundance trajectories. Though we are confident in our identification to the family level, we could not identify the larval nematodes we detected at the species level, so it is possible that parasites of an increasing species tend to infect pink and chum salmon, while parasites of a stable species tend to infect coho and sockeye. While past studies have found similar parasite species among these hosts (Deardorff & Kent, 1989; Karl et al., 2011; Myers, 1979), multiple species of anisakids have been detected in Alaskan salmon (specifically *Anisakis* spp., *Pseudoterranova decipiens*, and *Pseudoterranova* spp.). *Anisakis* spp. is increasing in abundance globally while *Pseudoterranova* spp. is remaining largely stable (Fiorenza, Wendt, et al., 2020; Mastick, Fiorenza, et al., 2024). It is therefore possible that the trends we detected reflect multiple anisakid species, and one species that is transmitted through chum and pink salmon is increasing over time, while another species infecting sockeye is not.

### 4.2 Implications

That we observed an increase in anisakids among Alaskan pink and chum salmon suggests these parasites are on the rise in commercially and ecologically important salmon species. Anisakids, if consumed alive, can cause anisakidosis in humans (Measures, 2014), which generally results in symptoms that resemble food poisoning (Bouree et al., 1995; Deardorff et al., 1991). Anisakids are sensitive to extreme temperatures and can be killed by freezing or cooking the fillet (Buchmann & Mehrdana, 2016), therefore, anisakids will be killed by the canning process. In addition, preparing salmon according to U.S. FDA guidelines will kill any anisakids present. However, consumers who have been previously exposed to anisakids can develop sensitivities to anisakid antigens (Alonso‐Gómez et al., 2004). Allergens from anisakids can remain in the fish product even if the worms are dead and cause allergic reactions to sensitized consumers (Audicana et al., 1997), though this is true for all methods of cooking infected fish. An increase in these parasites could result in more instances of these allergic reactions in sensitized consumers.

From an ecosystem perspective, this increase in anisakids may result in higher parasite prevalence across a suite of hosts, including salmon, but it can also be considered an indication of ecosystem recovery from historical exploitation. Parasites can be used as a proxy of ecosystem health, as abundant and healthy host populations are necessary to support parasite populations (Hudson et al., 2006; Lafferty, 2008). The presence of these nematodes indicates that their requisite hosts are abundant enough for the completion of their life cycles (Hudson et al., 2006; Lafferty, 2008). We do not have enough temporal data to say what anisakid burden in salmon might have been over a century ago, prior to European colonization, commercial fishing, and whaling in this region. But in other regions, increases in anisakids have reflected a return to historical abundances that were driven by the high availability of marine mammal definitive hosts in pre‐industrial ecosystems (Mastick, Welicky, et al., n.d.). The previous decline of marine mammals in the region due to commercial whaling and fur pelt industries may have bottlenecked anisakid populations, and the rise we observe here may merely reflect a return to more natural conditions.

Although the increase in anisakids that we observed might represent a return to more “natural” conditions, that does not mean that it is an unmitigated good. While anisakids may be returning to a previous baseline, their hosts face different threats than they did historically. Alaskan salmon face numerous threats that could impact populations, including increasing stream temperatures (Bryant, 2009; Mauger et al., 2017; Shanley et al., 2015), increasing precipitation and streamflows (Shanley & Albert, 2014; Sloat et al., 2017), invasive species and range expansions of competitor species (Jalbert et al., 2021), and landscape change (Schoen et al., 2017). Similarly, though marine mammals in this region are recovering, they face numerous other threats, including vessel collisions (Schoeman et al., 2020) and noise (Erbe et al., 2019), fisheries interactions and bycatch (Read, 2008; Read et al., 2006), climate change (Learmonth et al., 2006), accumulation of pollutants (Reijnders & de Ruiter‐Dijkman, 1995), and entanglement and ingestion of man‐made materials (Poeta et al., 2017). Populations that face multiple threats may be particularly vulnerable to increases in parasite burden, as the impacts of these threats can act synergistically (Kellar et al., 2017; Marcogliese, 2008; Simmonds, 2018; Wright, 2012). The increase in anisakids that we observed suggests that certain species of salmon and marine mammals in this region are more likely to become infected than they were 40 years ago, which may be a cause for concern for endangered or at‐risk Alaskan salmon and marine mammals.

### 4.3 Caveats

Chilling practices are intended to preserve catches longer, and a co‐benefit of this practice is that it may reduce the migration of nematodes into the fillet of the fish. However, we found that chilling practices did not have a significant effect on parasite abundance in salmon cans. This could be due to the temperature of refrigeration. Cipriani et al. (2016) showed that anisakid abundance in the fillet is lower when stored at a low temperature (2°C), as migration of worms into the fillet increases after capture when stored at a higher temperature (5 or 7°C). Salmon in Alaska are chilled between −2.2–2.2°C, so anisakids should be prevented from migrating into the fillet, resulting in fewer worms than in unrefrigerated fish. However, chilling practices are not always strictly enforced (Bruce Odegaard, Seafood Products Association, pers. comm.). Additionally, we used the pre‐ and post‐1995 time frame as an estimate, but there was not a specific date when every fishing vessel began chilling their fish. So, it is perhaps more likely, because not every vessel chills their catch and because the implementation of onboard chilling occurred over an unknown time frame, that our chilling metric did not effectively capture the nuanced differences in vessel chilling practices across vessels. However, if there were an effect of chilling that we were not able to detect in this study, the increase in anisakid abundance over time may be an underestimate of the true increase in anisakid burden—that is, had the chilling effect not been implemented in the middle of our time series, the observed increase in anisakids might have been much greater.

Though we demonstrated that parasite detection was possible in canned salmon, we have no validation study to confirm that the detectability of nematodes is not affected by can age. Canned salmon has a commercial shelf life of 5 years, and the oldest cans in our collection were over 40 years old. Older cans were found to degrade over time—cans bulged at the seams, formed black blisters known as “sulfite blackening” along the inside, fizzed when opened, and showed visible signs of rot in the canned fillet. Rot included cloudy liquid within the can, softened fillets, and green, glassy cyst‐like orbs around the spine that are likely attributable to chemical reactions inside the can over time. It is possible that the trends we detected in anisakid burden were due to degradation with can age, and that more worms were detectable in recent cans because they had had less time to decay. However, we believe that this is unlikely for a few reasons. First, we do not expect that nematodes would have degraded differently across the salmon species after being canned, as they were all treated to the same thermal processing procedure and storage. If the patterns we detected were due to worms degrading over time, we would expect to have seen the same rate of degradation across the four salmon species. Second, we never detected any material that resembled part of a nematode; we only ever found whole nematodes. If anisakids were degrading in the cans over time, we would have expected to detect some partially degraded nematodes. Finally, anisakid cuticles consist of multiple layers that allow them to withstand both varying environmental conditions and the acidic environment of a host's gastrointestinal tract (Myers, 1960; Page, 2013). This also makes them resistant to degradation over time. For example, although anisakids have not been reported in archeological studies (Arriaza et al., 2010), other parasitic roundworms have been found during examination of coprolites that were thousands of years old (Reinhard & Araújo, 2012; Reinhard & Bryant Jr., 2008). We cannot rule out the possibility that the pattern reported here is an artifact of nematode degradation with time, but we believe that this is unlikely.

It is possible that the selection of fillets for canning changed over time, influencing the worm burden. For example, if processors became more likely over time to divert wormy fillets to canning, this shift could have produced a spurious increasing trend in anisakid burden. While canning in this region has become less popular over the duration of our study (e.g., 30–40 canneries operated in Alaska in the 1970s, but there were only 16 in 2023; John Daly, industry expert, pers. comm.), the poorer quality fish, classified as No. 3 grade, were always selected for canning (John Daly, industry expert, pers. comm.). Poor quality could include softer or bruised fish, seal bites, blemishes, older catches (i.e., more time on ice), or smaller body sizes. The specifications for a No. 3 grade vary slightly from company to company but have not changed systematically over the study period (John Daly, industry expert, pers. comm.), therefore the quality of fish canned over the study period was unlikely to have varied. Selecting progressively older fish could also contribute to a more parasitized can, as parasite burden generally increases with fish length (Poulin, 2000), however, this is unlikely, as average body size has been declining in North Pacific salmon (Bigler et al., 1996; Oke et al., 2020). Additionally, through our conversations with salmon canning industry professionals, we have learned that the selection of salmon for cans varies more from company to company than over time, which would only result in a trend over time if every cannery changed its practices in the same way over time. If the demand for canned salmon was much greater at the start of our study period, then a greater proportion of all fish caught may have gone to canning, regardless of condition or size, which would have resulted in a concentration of the most heavily infected fish in canning during later years with lower demand. However, that does not appear to be the case — demand was low in the 1970s and canned salmon markets grew from 1980 to the 1990s (U.S. National Park Service, 2023). Therefore, it is unlikely that changes in salmon selection over time resulted in the observed trend of increasing anisakids in canned salmon.

## 5 CONCLUSION

We used a novel data source—archived canned salmon—to detect an increase in anisakid nematodes in Alaskan chum and pink salmon. We did not detect similar trends in sockeye or coho. The increasing burdens of anisakids in these two species are probably attributable to increasing marine mammal abundance in this region, though climate change may also contribute by speeding up anisakid growth and reproduction.

## AUTHOR CONTRIBUTIONS

**Natalie Mastick:** Conceptualization (equal); data curation (equal); formal analysis (lead); investigation (equal); methodology (equal); project administration (lead); writing – original draft (lead). **Rachel Welicky:** Conceptualization (equal); formal analysis (supporting); investigation (supporting); methodology (equal); writing – review and editing (equal). **Aspen Katla:** Investigation (equal); writing – review and editing (equal). **Bruce Odegaard:** Conceptualization (supporting); resources (lead); writing – review and editing (equal). **Virginia Ng:** Conceptualization (supporting); resources (supporting); writing – review and editing (equal). **Chelsea L. Wood:** Conceptualization (equal); methodology (equal); supervision (lead); writing – review and editing (equal).

## CONFLICT OF INTEREST STATEMENT

The authors declare no competing interests.

## Supporting information


Appendix S1.

Tables S1‐S2.

## Data Availability

Data generated in this study and code used for analysis have been uploaded as supporting materials for review. Upon publication, the data and corresponding analysis code will be made available via a GitHub repository: https://github.com/wood‐lab/Mastick_et_al._EcoEvo
