# Supplementary material for: Opening a can of worms: Archived canned fish fillets reveal 40 years of change in parasite burden for four Alaskan salmon species
Source: Ecol Evol. 2024 Apr 4;14(4):e11043. doi: 10.1002/ece3.11043 (PMC10994144; doi:10.1002/ece3.11043)
Supplement: Supplementary file 1 — Appendix S1. [file ECE3-14-e11043-s002.docx]

APPENDIX S1

Testing methods for detecting nematodes in canned fish fillets

Using cans of unknown origin dates or species contents, we tested three methodologies of dissecting fillets: the UV-Press method (Karl and Leinmann 1993), the candling method (Valdimarsson et al. 1985), and manual dissection with forceps. The UV-Press method is commonly used to detect anisakids in fresh or frozen fillets, in which a fillet is either flattened between two acrylic sheets or vacuum-pressed to 2-3 mm thick (Karl and Leinmann 1993; Gomez-Morales et al. 2018). A 366 nm UV light is shined at the flattened fillet in a darkened room and the nematodes fluoresce and can easily be counted (Gomez-Morales et al. 2018). The candling method involves placing a fillet (with skin removed) on a backlit surface and examining the fillet for coiled nematodes (Valdimarsson et al. 1985). Thinly slicing the fillets or pressing the fillets to 3-4 mm thickness before candling can improve nematode detectability (Karl and Leinmann 1993). To test the UV-Press and candling methods, we pressed a section of the canned fillet known to have at least one nematode present between two plexiglass plates using an industrial press, flattening the fillet to a thickness of 2-3 mm. When subjected to a UV light, the nematodes did not fluoresce, possibly due to degradation of proteins during cooking. When candled, worms were detectable, but the high moisture content of the can and the softness of the cooked fillet made it difficult for the nematode’s position to be maintained when the plates were separated, as the flattened muscle came apart once the top plexiglass plate was removed. We tested dissecting each fillet manually with forceps, and found that this was the cleanest, most effective way to detect, count, and preserve nematodes without damaging the parasite. We found that the nematodes formed pockets in the cooked fillet that were detectable when using forceps, but that were not retained when the fillet was pressed. Therefore, we used the forceps dissection method for all cans.

We developed our search image on spare cans, during which time we had a second observer check each fillet for any remaining nematodes. Once our dissectors reached 100% agreement, we began dissecting our sample cans. Dissections were conducted by one trained observer (AK or NM), and a second observer checked cans at random. We opened each can and noted the can size as a metric of fillet mass. If the fillet appeared mostly intact, we then drained the can of most of its liquid. If the fillet appeared to have degraded into the liquid, we did not drain the can prior to examining the fillet. We scooped a portion of the fillet onto a clear acrylic sheet atop a black benchtop to maximize contrast. Using two pairs of forceps (4.25-inch, fine point), we carefully dissected the fillet into small pieces (less than 1 cm^2^). When we came across a nematode pocket, we carefully extracted the nematode from the fillet and placed it in a vial of 70% ethanol for preservation. We tallied the number of worms extracted for each can.

APPENDIX S1 REFERENCES

Gómez-Morales, M.A., Castro, C.M., Lalle, M., et al. 2018. UV-press method versus artificial digestion method to detect Anisakidae L3 in fish fillets: Comparative study and suitability for the industry. *Fisheries Research* 202:22-28.

Karl, H. & Leinemann, M. 1993. A fast and quantitative detection method for nematodes in fish fillets and fishery products. *Arch. Lebensmittelhyg* 44: 124-125.

Valdimarsson, G., Hjalti, Einarsson, H. & King, F.J. 1985. Detection of parasites in fish muscle by candling technique. *Journal of the Association of Official Analytical Chemists* 68:549-551.
