# Supplementary material for: Opening a can of worms: Archived canned fish fillets reveal 40 years of change in parasite burden for four Alaskan salmon species
Source: Ecol Evol. 2024 Apr 4;14(4):e11043. doi: 10.1002/ece3.11043 (PMC10994144; doi:10.1002/ece3.11043)
Supplement: Supplementary file 2 — Tables S1‐S2. [file ECE3-14-e11043-s001.docx]

APPENDIX S2

Table S1: Candidate models for Model 1, assessing change in anisakids for chum, coho, and pink salmon. All models included random effects of factory and company nested within region. The model used for analysis is represented in bold.

| Model | AIC | ΔAIC |
| --- | --- | --- |
| **Anisakid count ~ can size + chilling practice + year*salmon species** | 372.2 | 0.3 |
| Anisakid count ~ can size + year*salmon species | 371.9 | 0 |
| Anisakid count ~ can size + chilling practice + year + salmon species | 373.8 | 1.9 |
| Anisakid count ~ can size + chilling practice + year | 384.6 | 12.7 |
| Anisakid count ~ can size + chilling practice + salmon species | 387.4 | 15.5 |

Table S2: Candidate models for Model 2, including sockeye-only. All models include the random effects of factory, company, and region interacting with time period. The model used in the analysis is represented in bold.

| Model | AIC | ΔAIC |
| --- | --- | --- |
| Anisakid count ~ can size + year + chilling practice | 211.0 | 2.0 |
| Anisakid count ~ can size + year | 209.0 | 0 |
| Anisakid count ~ can size + chilling practice | 209.8 | 0.8 |
| Anisakid count ~ can size | 209.3 | 0.3 |
